# As a Staple Food Substitute, Oat and Buckwheat Compound Has Health-Promoting Effects for Diabetic Rats

**DOI:** 10.3389/fnut.2021.762277

**Published:** 2021-12-24

**Authors:** Siqi Liu, Xueqian Yin, Chao Hou, Xinran Liu, Huijuan Ma, Xiaoxuan Zhang, Meihong Xu, Ying Xie, Yong Li, Junbo Wang

**Affiliations:** ^1^Department of Nutrition and Food Hygiene, School of Public Health, Peking University, Beijing, China; ^2^Department of Pharmaceutics, School of Pharmaceutical Sciences, Peking University, Beijing, China

**Keywords:** oat, buckwheat, dietary intervention, staple food, diabetes, glucose metabolism, gut microbiota

## Abstract

Dietary intervention is crucial for the prevention and control of diabetes. China has the largest diabetic population in the world, yet no one dietary strategy matches the eating habits of the Chinese people. To explore an effective and acceptable dietary pattern, this study uses oat and buckwheat compound (OBC) as a staple food substitute and explored its effects on diabetic Sprague–Dawley rats. The model of diabetic rats was established by combining high-calorie feed and streptozotocin (STZ) injection. The dietary intervention for the seven groups, including a normal control group, a model control group, a metformin control group, a wheat flour control group, and three OBC groups with different doses, started from the beginning of the experiment and lasted for 11 weeks, two consecutive injections of STZ in small doses were operated at the 6th week. General states, glucose metabolism, and lipid metabolism indexes were measured. Antioxidant and inflammatory indexes and pathologic changes of kidney and liver tissues were tested. Changes in kidney and ileum ultramicrostructure were detected. What's more, ileal epithelial tight junction proteins and gut microbiota were analyzed. Significant decreases in fasting blood glucose (FBG), glucose tolerance, serum insulin, and insulin resistance were observed in rats intervened with OBC, and these rats also showed a higher level of superoxide dismutase (SOD) together with improved lipid metabolism, attenuated inflammation, and liver and kidney injuries. In addition, in OBC groups, the intestinal barrier was improved, and the disturbance of gut microbiota was reduced. These results suggest that OBC has health-promoting effects for diabetic rats, and since oat and buckwheat are traditionally consumed grains in China, OBC could be a potential and easy-to-accept staple food substitute for the dietary pattern for Chinese.

## Introduction

Diabetes is a serious chronic metabolic disease with complications in many parts of the body and can increase the overall risk of dying prematurely. Around 463 million people are estimated to be suffering from diabetes in 2019, which is expected to rise to 700 million (10.9%) by 2,045 (1). The prevalence of diabetes in China is particularly serious. According to the International Diabetes Federation Diabetes Atlas 9th Edition, there are about 116 million diabetic patients in China, and 147 million are expected by 2,045 [International Diabetes Federation (IDF) Diabetes Atlas 2019].

According to the WHO, a healthy diet plays a key role in reducing the risk of diabetes. Several dietary patterns such as the Mediterranean diet or patterns that replace all or part of the staple food with substances like potatoes or nuts have been demonstrated to reduce diabetes risk (2). However, the dietary preferences of Chinese people are quite different from Western countries. Grains account for a large part of the diet as the main staple food. As the increasing evidence revealed the predisposition to insulin resistance and diabetes due to traditional staple consumption of rice and wheat (3), oats and buckwheat, widely consumed grains in China, are better staple food substitutes. Oat and buckwheat had been proved to have significant effects on controlling hyperglycemia (4), insulin response (5), and diabetic kidney disease (6). In 2016, by taking a randomized control trial (RCT), Li (7) and others concluded that both short and long term oat intake (at least 50 g oats per day) had a significant effect on controlling hyperglycemia; similarly, another RCT showed that buckwheat intake by 110 g/d could also improve insulin resistance in patients with type 2 diabetes mellitus (8). What's more, various research have shown that ingredients of oat as oat oligopeptides and oat β-glucan can exert a hypoglycemic effect (9, 10), as well as rutin and d-chiro-inositol in buckwheat could improve insulin sensitivity and regulate serum glucose level (11, 12). In addition, the correlation between diabetes and gut microbiota disorder has attracted increasing attention, and certain whole grains such as oat and barley, which are rich in dietary fibers, have also been shown to promote and maintain gut microbiota (13). However, due to the distinctive taste and texture, it is hard for people to consume one certain grain daily. By mixing two grains, the palatability could be improved. What's more, as single grains are usually not suitable as a staple food due to the lack of certain nutrients, especially amino acids, by mixing buckwheat and oat, amino acids could be complementary between buckwheat and oat, as buckwheat contains more lysine and arginine, and oat contains more glutamic acid, proline, and leucine.

In this work, we used oat and buckwheat compound (OBC) as a staple food substitute and explored its effects on diabetes by researching the physiology and gut microbiota of diabetic rats. This work will provide a valuable reference for promoting the exploration of dietary patterns that are suitable for the prevention and treatment of diabetes in China.

## Materials and Methods

### Materials and Reagents

Oat and buckwheat compound is a mixture made from buckwheat and oat mixed in the ratio 2.2 to 1. The ratio of buckwheat to oat was selected by our previous experiments. Both oat (Bayou 1) and buckwheat (Dasanleng) were purchased from DongFangLiang Life Technology Ltd. (Shanxi, China). The nutrients of oat and buckwheat used in the experiment are shown in Table 1, tested by the College of Food Science and Nutritional Engineering, China Agricultural University.

**Table 1:** Nutrients of buckwheat and oat.

**Nutrients**	**Buckwheat**	**Oat**
Energy (kJ/100 g)	1,407	1,574
Protein (g/100 g)	11.9	13.9
Fat (g/100 g)	2.8	8.0
Carbohydrate (g/100 g)	61.8	58.3
Total dietary fiber (g/100 g)	6.37	6.36
Ash (g/100 g)	1.6	1.5
Water (g/100 g)	15.5	11.9
Vitamin E (mg/100 g)	7.08	1.22
Phosphorus (mg/100 g)	344	327
Kalium (g/kg)	4.36	3.54
Manganese (mg/kg)	12.6	40.4
Selenium (mg/kg)	0.0361	0.0311
Quercetin (g/mg)	<0.1	<0.1
Ferulic acid (μg/100 g)	1.01	29.9
Protocatechuic acid (μg/100 g)	14.6	10.6
Vanillic acid (μg/100 g)	<0.4	3.59

Streptozotocin (99.8% purity) was purchased from Sigma Chemical Co. (St Louis, MO, USA). Glucose (99.8% purity) was purchased from Beijing Jinhua Taiya Chemical Reagent Co., Ltd. (Beijing, China). Metformin hydrochloride extended-release tablets were purchased from Sino-American Shanghai Squibb Pharmaceuticals Ltd. (Shanghai, China, National medicine permit number: H20023370). Citric acid (99.9% purity) and sodium citrate (99.9% purity) used in the streptozotocin injection solution were purchased from Beijing Tong Guang fine chemicals company.

Basic feed was processed by Beijing Biotech HD Biotechnology Co., Ltd. (Beijing, China), according to the national standard (GB 14924.3-2010). High-calorie feed was processed by Beijing Biotech HD Biotechnology Co., Ltd. (Beijing, China) according to the evaluation method of auxiliary hypoglycemic function of health food (National Food and Drug Administration [2012]107). Feed for OBC groups and wheat flour control (WFC) group were made by replacing the cornstarch in high-calorie feed with different proportions of OBC or wheat flour (OBC content in each group feed: low dose: 29.3 g/kg, medium-dose: 58.7 g/kg, high dose: 117.3 g/kg). At the same time, maltodextrin, 10 in each group, had been adjusted to ensure that the feed quality of each group was equal, while calories of feeds for each group were in agreement (the differences were <0.07%). The feed ingredients of each group are shown in Table 2.

**Table 2:** Feed ingredients of each group (g/kg).

**Group**	**Cornstarch**	**Wheat flour**	**OBC**	**Maltodextrin**	**Sucrose**	**Soybean oil**	**Lard**
NC	298.6	0.0	0.0	33.2	331.7	23.7	19.0
MC	84.8	0.0	0.0	116.5	201.4	29.1	206.8
WFC	0.0	159.3	0.0	42.1	201.4	29.1	206.8
MPC	84.8	0.0	0.0	116.5	201.4	29.1	206.8
OBC-L	45.0	0.0	39.9	116.5	201.4	29.1	206.8
OBC-M	5.1	0.0	79.7	116.5	201.4	29.1	206.8
OBC-H	0.0	0.0	159.3	42.1	201.4	29.1	206.8

*NC, normal control group; MC, model control group; WFC, wheat flour control group; MPC, metformin positive control group; OBC-L, low-dose group; OBC-M, medium-dose group; OBC-H, high-dose group*.

### Animal Housing Conditions

Male Sprague–Dawley rats (200 ± 10 g) were provided by the Department of Laboratory Animal Science, Peking University (Beijing, China) and bred in a specific pathogen free (SPF) room there, production certificate No.: SCXK (Beijing) 2016-0010, use license No.: SYXK (Beijing) 2016-0041. Two rats were housed per cage, with the temperature of the SPF room controlled at 25°C ± 1°C, relative air humidity controlled at 50–60%, and 12-h light/dark cycles. Due to the polyuria caused by diabetes, the cage bedding was changed daily after injecting STZ. All animal procedures meet the Regulation on the Administration of Laboratory Animals and were approved by the Ethics Committee of Peking University (ethics No.: LA2018189).

### Establishment of Diabetes Model

Healthy adult male SD rats were fed adaptively for 1 week before the experiment. After 45 days fed by each group's corresponding feed, rats to be modeled received two intraperitoneal injections of low-dose STZ (30 mg/kg body weight) at an interval of 1 week. Three days after the second injection, rats were fasted for 5 h to measure their tail vein blood glucose. Rats with a blood glucose of 10–25 mmol/L were used as diabetic rats for our study.

### Experimental Design

**Figure 1:**
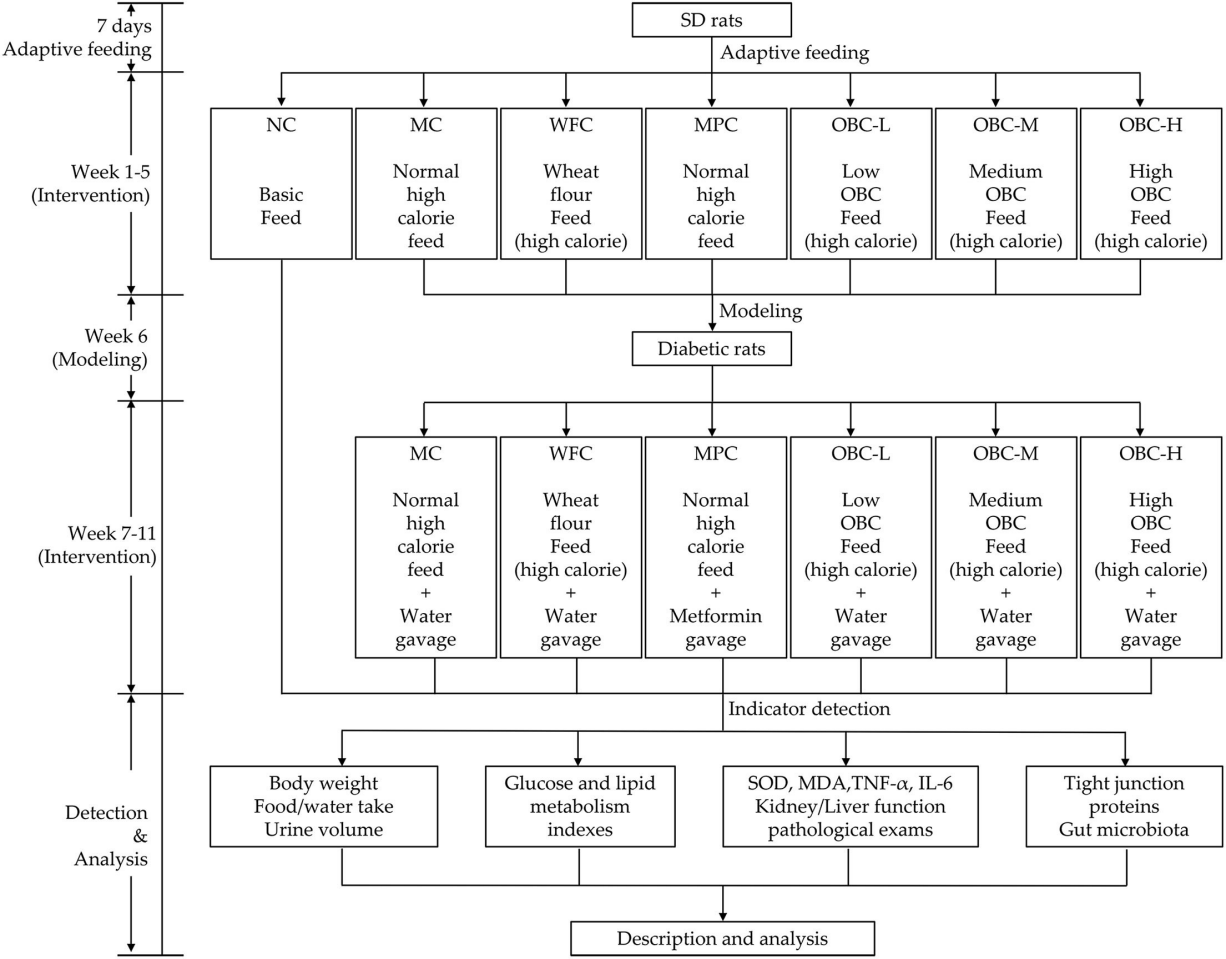
Schematic diagram of the study protocol.

Based on their baseline serum glucose, 72 rats were randomly divided into seven groups, the normal control group (NC, *n* = 10), the model control group (MC, *n* = 10), the (WFC, *n* = 10), the metformin positive control group (MPC, *n* = 10), and three OBC treatment groups including a low dose group (OBC-L, *n* = 10), a medium dose group (OBC-M, *n* = 11), and a high dose group (OBC-H, *n* = 11). As a reference hypoglycemic drug, metformin was used to intervene rats in the MPC group by gavage after the injection of STZ. The initial dose of metformin was 50 mg/kg body weight, and it increased by 50 mg/kg body weight every 2 weeks until the maximum dose at 200 mg/kg body weight. All rats in other groups received distilled water every day at the same time with a bodyweight volume of 10 ml/kg until the end of the experiment. The general state of rats includes haircoat state, mental state, body weight, water, and food intake were recorded daily. Six rats were randomly selected in each group and fed individually in the metabolic cage for 24 h to collect and record the urine volume of the rats. Each metabolic cage has a funnel and measuring cylinder for urine collection. The schematic diagram of the study protocol is shown in Figure 1.

### Determination of Fasting Blood Glucose and Oral Glucose Tolerance Test

Fasting blood glucose levels of rats were measured every week. Rats fasted for 5 h before the FBG was measured through the tail vein. The oral glucose tolerance test was done on the 3rd, 7th, and 11th weeks of the experiment. After 12 h of fasting, the blood glucose of rats was measured by tail vein as the blood glucose level at 0 h, and then rats were gavaged with 50% glucose solution at a dose of 2 g/kg body weight, and blood glucose was recorded 0.5, 1, and 2 h after gavage. The area under the blood glucose curve of oral glucose tolerance test (OGTT-AUC) was calculated by the following formula:


$$\begin{array}{l} OGTT-AUC=0.25\times 0\;hFBG(mmol/L)+0.5\\ \;\times 0.5hFBG\;(mmol/L)+0.75\\ \;\times 1hFBG(mmol/L)+2hFBG(mmol/L) \end{array}$$

Homeostasis model assessment of insulin resistance (HOMA-IR) was calculated by the following formula:


$$HOMA-IR=FBG(mmol/L)\times \frac{INS(\mu IU/mL)}{22.5}$$

### Biochemical Analysis and Pathological Examination

The main organs (kidney, liver, ileum, and caecum) of rats were removed and weighed at the end of the experiment to calculate the organ coefficients and for further testing including hematoxylin–eosin (HE) staining and ultramicrostructure observation. The level of serum insulin was detected by using an XH-6080 γ radioimmunocounter (Zhonghe, Xi'an, China). The level of SOD and malonaldehyde (MDA) were detected by using a 721-G Visible spectrophotometer (Jingke, Shanghai, China). The level of FBG and all other biochemical indicators include glycated serum protein, tumor necrosis factor-α (TNF-α), interleukin-6 (IL-6), aspartate aminotransferase (AST), and alanine aminotransferase (ALT) were detected by using an AU480 automatic biochemistry analyzer (Olympus, Tokyo, Japan). The pathologic changes of kidney and liver tissues were detected by HE staining. Kidney ultramicrostructure was observed with a transmission electron microscope. Western blotting was used to detect ileal tight junction proteins. Cecal feces sample microbiota analysis was divided into the following sections: sample collection, DNA extraction and amplification, 16S rDNA sequencing, and taxonomic classification.

### Statistical Analysis

Data accorded with normal distribution and homogeneity of variance were expressed as means ± SD, compared by one-way analysis of variance (ANOVA), otherwise, expressed as medians and quartiles, and compared by Kruskal–Wallis H test. SPSS 22.0 (IBM Corp, Armonk, NY, USA) was applied for statistical analysis. The value *p* < 0.05 was considered statistically significant.

## Results

### Effect of OBC on General State and Bodyweight

**Figure 2:**
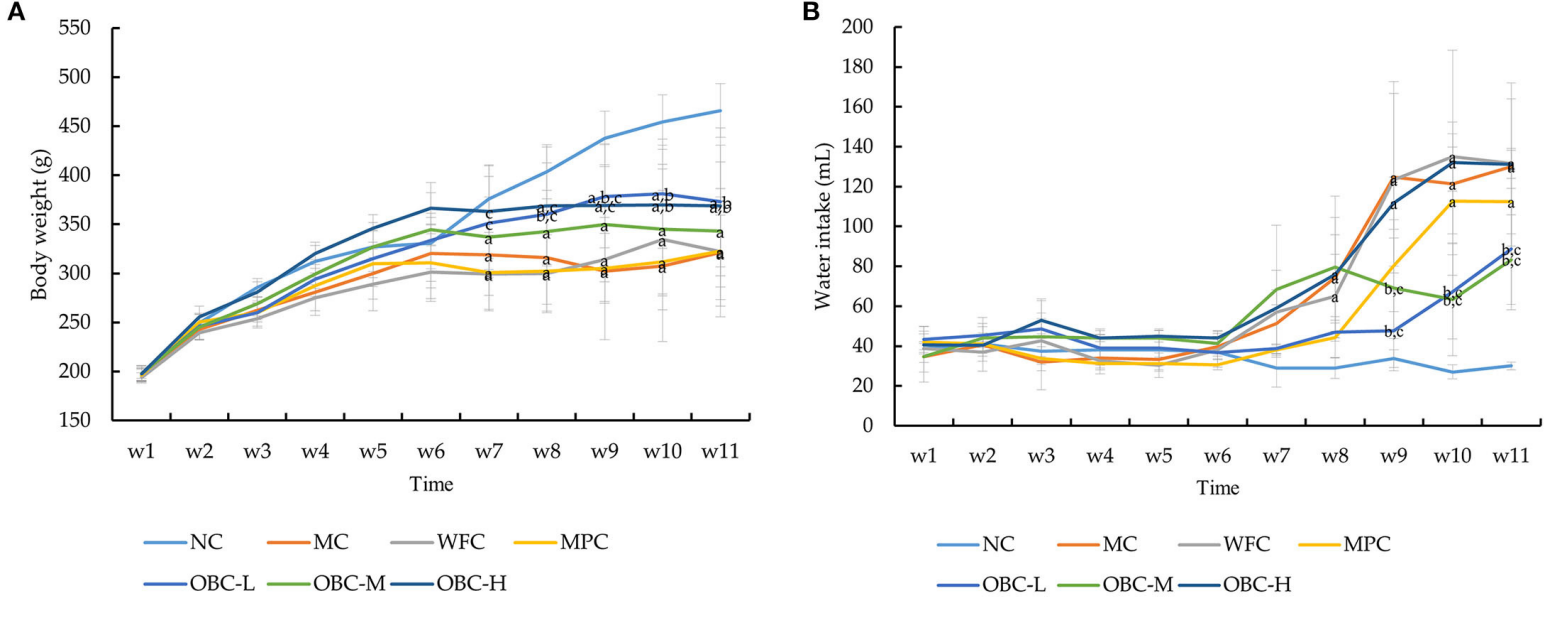
Effect of OBC on body weight **(A)**, water intake **(B)**. NC (*n* = 10), MC (*n* = 9), WFC (*n* = 9), MPC (*n* = 8), OBC-L (*n* = 8), OBC-M (*n* = 10), OBC-H (*n* = 9), values are presented as mean ± SD. (a) *p* < 0.05 vs. NC rats, (b) *p* < 0.05 vs. MC rats, (c) *p* < 0.05 vs. WFC rats.

With withered yellow fur and less well-formed feces, the diabetic rats showed a much poorer haircoat quality and a symptom of diarrhea, compared with normal rats. Besides, rats with STZ-induced diabetes also shared reduced general activity. As shown in Figure 2A, there were no significant differences among the seven groups of body weight before modeling. After the injection of STZ, compared with the normal group, the body weight of rats in MC, MPC, WFC, and OBC-M groups significantly decreased from the 7th week, whereas the OBC-H group decreased later from the 8th week and the OBC-L group decreased from the 9th week. Compared with the MC group, the bodyweight of the OBC-L group was found markedly lower than that of the MC group from the 8th week and the OBC-H group in the 10th week. From the 7th week to the 9th week, the bodyweight of the OBC-L and OBC-H groups was significantly higher than that of the WFC group.

As shown in Figure 2B, compared with the normal group, water intake was found obviously increased in the MC and WFC groups from the 8th week. Water intake in the OBC-L and OBC-M groups was markedly lower than that in the MC and WFC groups from the 9th week. Food intake made no difference. This result suggests that OBC could improve diabetic symptoms in diabetic rats.

### Effect of OBC on Glucose Metabolism

**Figure 3:**
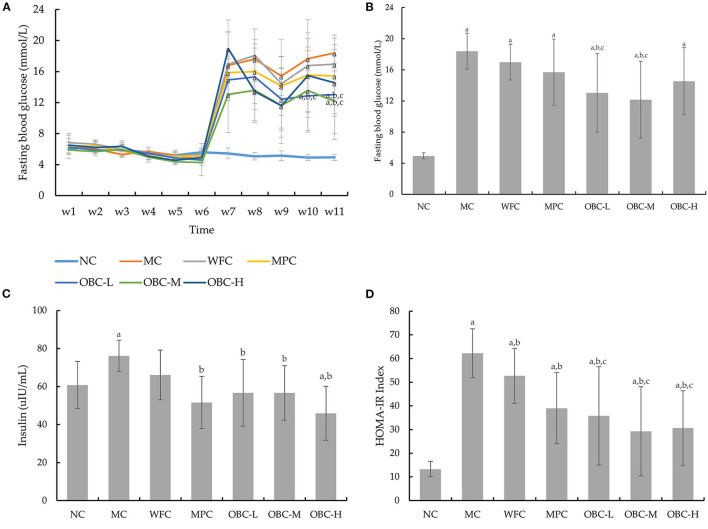
Effect of OBC on glucose metabolism: FBG from week 1–11 **(A)**, FBG in the 11th week **(B)**, serum insulin **(C)** and HOMA-IR **(D)**. NC (*n* = 10), MC (*n* = 9), WFC (*n* = 9), MPC (*n* = 8), OBC-L (*n* = 8), OBC-M (*n* = 10), OBC-H (*n* = 9), values are presented as mean ± SD. a *p* < 0.05 vs. NC rats, b *p* < 0.05 vs. MC rats, c *p* < 0.05 vs. WFC rats.

The fasting blood glucose of all rats showed a normal level (6.3 ± 0.91 mmol/L), and no significant differences were seen among these groups before modeling. After the diabetic model was established, different from the normal group, the FBG level statistically increased over time in the diabetic rats since the 7th week (Figure 3A). By the end of the treatment by OBC, rats in the OBC-L and OBC-M groups showed a significantly lower FBG level than rats in the WFC and MC groups (Figure 3B). This suggests that OBC could reduce fasting blood glucose in diabetic rats.

To analyze the effect of OBC on glucose tolerance in diabetic rats, we conducted the OGTT in the third, 7th, and 11th weeks. As shown in Table 3, there were no significant differences in OGTT-AUC among these groups before modeling, and after that, OGTT-AUC was found obviously increased in all diabetic rats in the 7th week. By the end of the experiment, rats treated with OBC showed some decreases of OGTT-AUC, among which, rats in the OBC-L group decreased statistically. These results suggest that OBC treatment may be conducive for the improvement of glucose tolerance in diabetic rats.

**Table 3:** Effect of OBC on OGTT-AUC.

**Group**	**OGTT-AUC (mmol/L)**		
	**3rd week**	**7th week**	**11th week**
NC	13.10 ± 0.75	12.68 ± 1.01	12.17 ± 0.75
MC	13.72 ± 0.98	45.60 ± 6.78^a^	46.44 ± 10.52^a^
WFC	13.62 ± 1.09	48.29 ± 6.95^a^	47.91 ± 7.77^a^
MPC	13.50 ± 1.46	42.76 ± 7.36^a^	46.42 ± 9.83^a^
OBC-L	13.20 ± 1.29	36.61 ± 14.70^a^	28.74 ± 13.56^a^, ^b^, ^c^
OBC-M	13.16 ± 0.98	43.17 ± 10.21^a^	41.38 ± 11.74^a^
OBC-H	14.05 ± 0.73	49.49 ± 5.92^a^	43.87 ± 11.99^a^

*NC, normal control group; MC, model control group; WFC, wheat flour control group; MPC, metformin positive control group; OBC-L, low-dose group; OBC-M, medium-dose group; OBC-H, high-dose group. Values are presented as mean ± SD*.

a *P <0.05 vs. NC rats*,

b *P <0.05 vs. MC rats*,

c *P <0.05 vs. WFC rats*.

As shown in Figure 3C, compared with normal rats, the insulin level in the MC group was found obviously increased. After the intervention of metformin and OBC, rats in MPC and OBC groups showed a significantly lower insulin level. The HOMA-IR index of all diabetic rats is higher than that of normal rats significantly, suggesting that insulin resistance in diabetic rats had occurred. After the treatment of wheat flour, metformin, and OBC, compared with the MC group, the HOMA-IR index of WFC, MPC, and OBC groups were all dramatically decreased by the end of the experiment. Meanwhile, diabetic rats treated with OBC showed a lower HOMA-IR index than rats treated with wheat flour markedly (Figure 3D).

### Effect of OBC on Blood Lipid Metabolism

**Figure 4:**
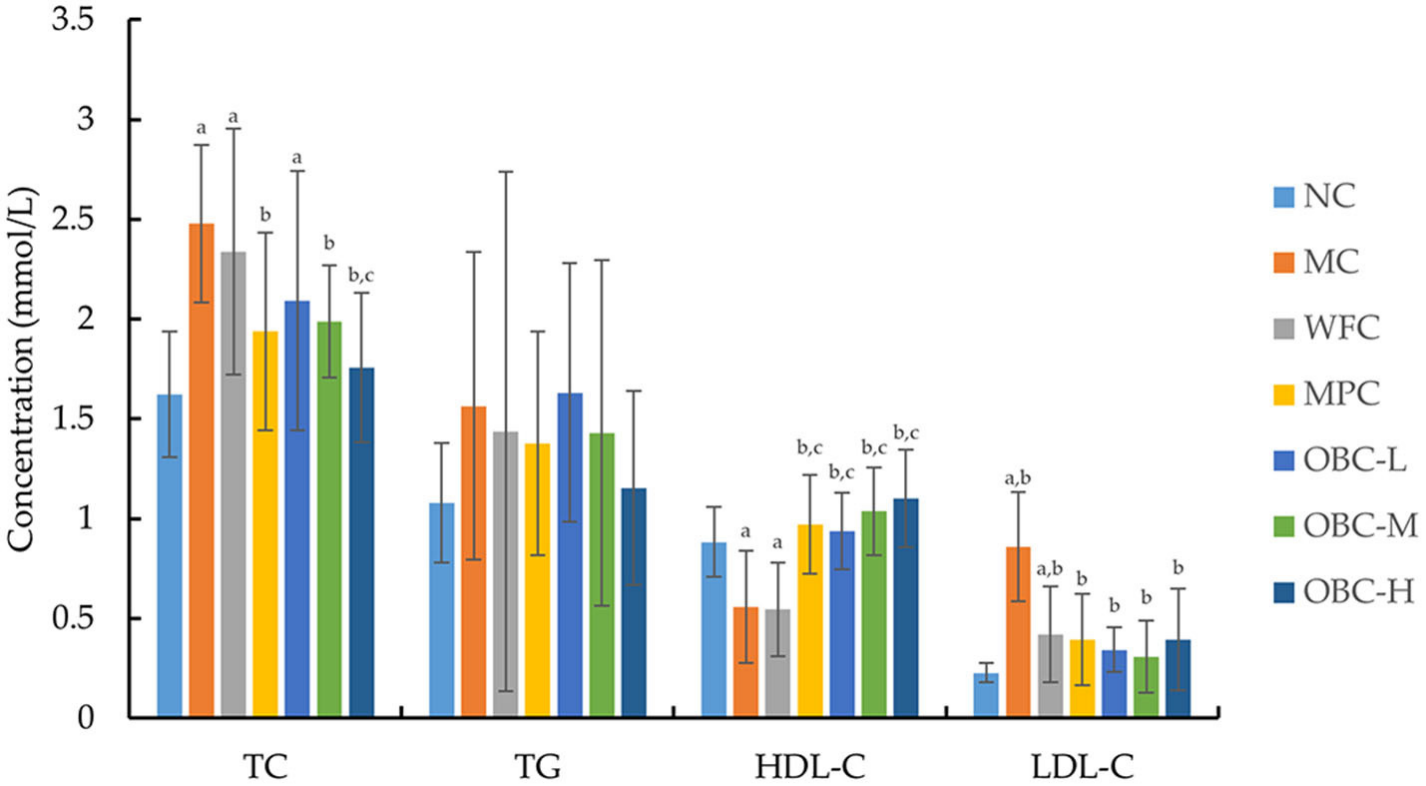
Effect of OBC on blood lipid metabolism. NC (*n* = 10), MC (*n* = 9), WFC (*n* = 9), MPC (*n* = 8), OBC-L (*n* = 8), OBC-M (*n* = 10), OBC-H (*n* = 9), values are presented as mean ± SD. a *p* < 0.05 vs. NC rats, b *p* < 0.05 vs. MC rats, c *p* < 0.05 vs. WFC rats.

As shown in Figure 4, the blood TC and LDL-C concentration of diabetic rats were significantly higher than that of normal rats, whereas the concentration of HDL-C was significantly lower. With the intervention of OBC, serum TC levels of rats in OBC-M and OBC-H groups were significantly lower than those in diabetic rats, serum HDL-C in all OBC groups increased significantly, and LDL-C decreased significantly, indicating that OBC could improve lipid metabolism. No significant differences showed in the TG level among these groups.

### Effect of OBC on Serum SOD and MDA

**Figure 5:**
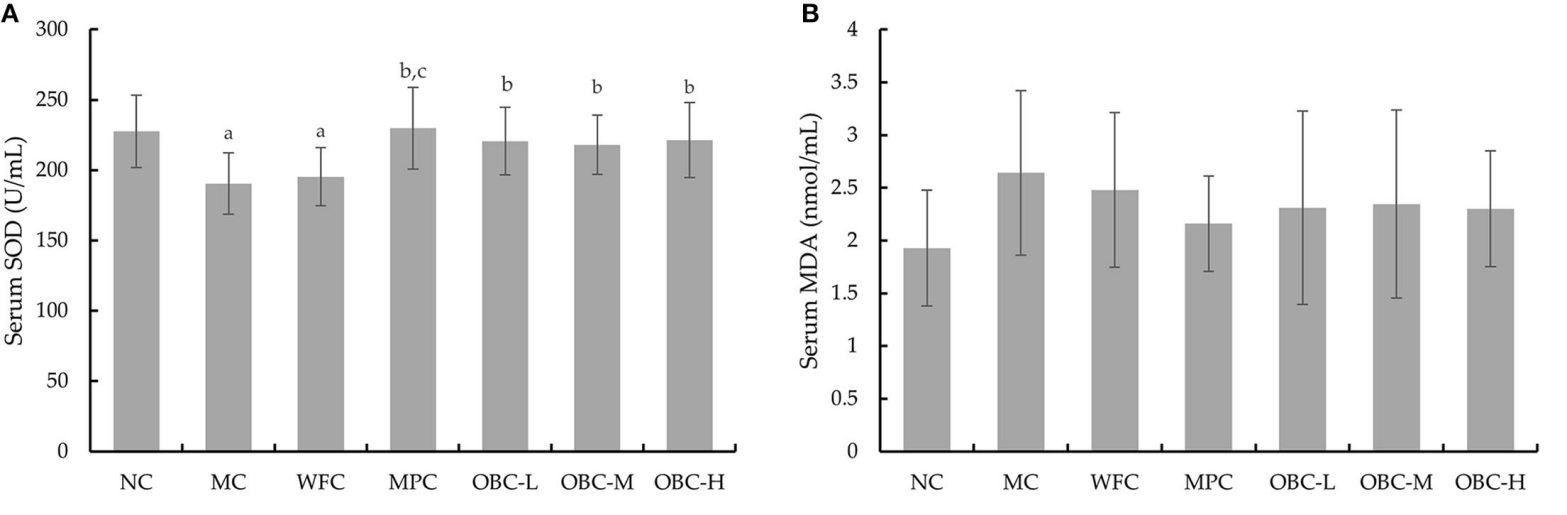
Effect of OBC on SOD **(A)** and MDA **(B)**.NC (*n* = 10), MC (*n* = 9), WFC (*n* = 9), MPC (*n* = 8), OBC-L (*n* = 8), OBC-M (*n* = 10), OBC-H (*n* = 9), values are presented as mean ± SD. a *p* < 0.05 vs. NC rats, b *p* < 0.05 vs. MC rats, c *p* < 0.05 vs. WFC rats.

The serum SOD level was significantly lower in MC and WFC groups as compared with normal rats; meanwhile, diabetic rats in OBC groups showed a markedly higher SOD level than rats in the MC group (Figure 5A), showing that OBC treatment could improve diabetic rat's antioxidant capacity. No significant differences were shown in the MDA level among these groups (Figure 5B).

### Effect of OBC on Serum TNF-α and IL-6

**Figure 6:**
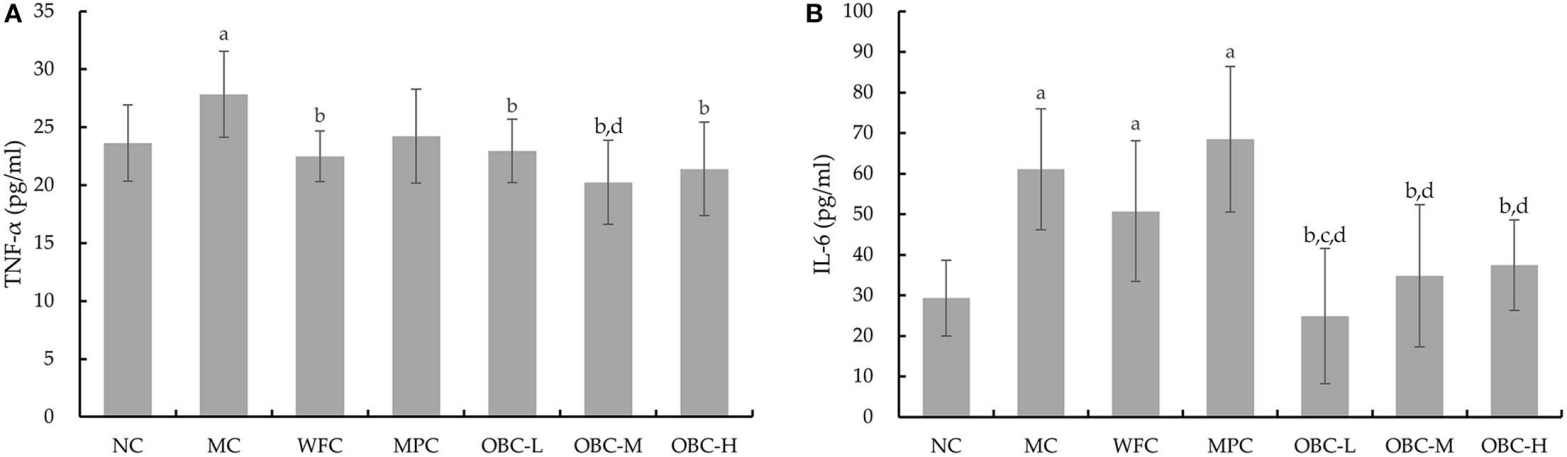
Effect of OBC on TNF-α **(A)** and IL-6 **(B)**.NC (*n* = 10), MC (*n* = 9), WFC (*n* = 9), MPC (*n* = 8), OBC-L (*n* = 8), OBC-M (*n* = 10), OBC-H (*n* = 9), values are presented as mean ± SD. a *p* < 0.05 vs. NC rats, b *p* < 0.05 vs. MC rats, c *p* < 0.05 vs. WFC rats, d *p* < 0.05 vs. MPC rats.

As shown in Figure 6, compared with the NC group, diabetic rats in the MC group showed increased serum TNF-α and IL-6. Rats in the WFC group and rats that intervened with OBC showed reduced serum TNF-α. The concentration of IL-6 in OBC groups was significantly lower than that in the MC group but IL-6 in the OBC-L group was statistically lower than the WFC group. Compared with rats interfered with metformin, the level of IL-6 in all OBC groups decreased, and TNF-α in the OBC-M group decreased.

### Effect of OBC on Liver Function

**Figure 7:**
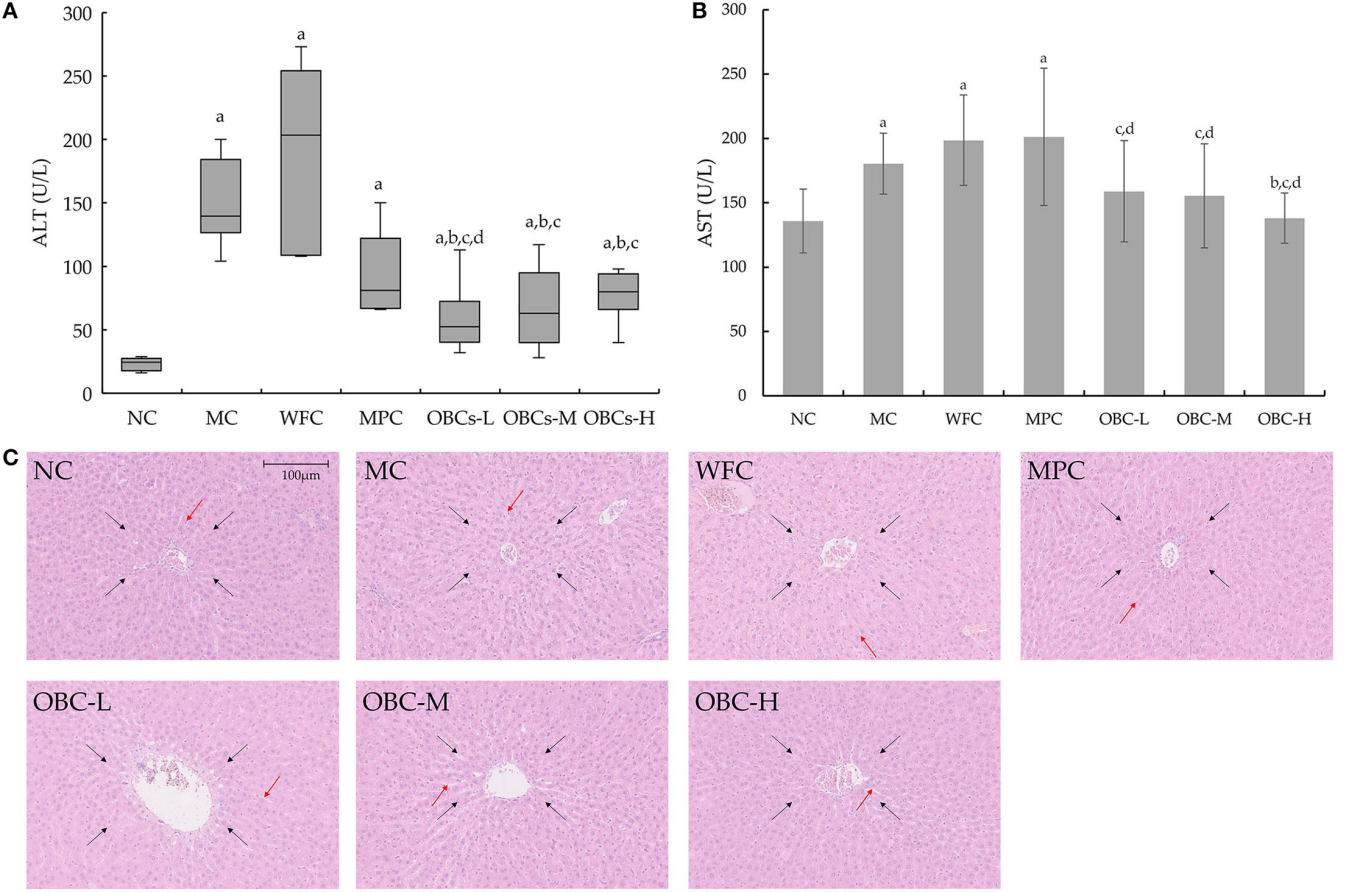
Effect of OBC on liver function. **(A)** ALT, **(B)** AST, **(C)** Pathological changes of liver in rats of each group. ALT of rats did not coincide with normal distribution, Kruskal-Wallis H test was applied for comparison of ALT. HE staining was used to examine liver tissues, black arrows indicate liver lobule, orange arrows indicate hepatic cords. All the images of liver histo-slides were taken at 200X from an Olympus microscope. NC (*n* = 10), MC (*n* = 9), WFC (*n* = 9), MPC (*n* = 8), OBC-L (*n* = 8), OBC-M (*n* = 10), OBC-H (*n* = 9), values are presented as mean ± SD. a *p* < 0.05 vs. NC rats, b *p* < 0.05 vs. MC rats, c *p* < 0.05 vs. WFC rats, d *p* < 0.05 vs. MPC rats.

Serum ALT and AST levels can reflect the degree of liver cell injury. The ALT data, which does not conform to the normal distribution, is represented by a boxplot and analyzed by Kruskal-Wallis H tests, showing that compared with rats in the NC group, serum ALT in diabetic rats increased significantly, and the ALT levels of rats in OBC groups were significantly lower than rats in the MC, WFC, and MPC groups (Figure 7A). The serum AST of rats in MC, WFC, and MPC groups were significantly higher than that of normal rats. The AST of rats in the OBC groups were significantly lower than that of the WFC group, whereas the AST of the OBC-H group was significantly lower than that of the MC group (Figure 7B). As shown in Figure 7C, rats in the NC group had the normal structure of hepatocytes with regular radical morphology of hepatic architecture with normal sinusoids. Diabetic rats in MC and WFC groups showed hepatocellular hypertrophy with disordered structure of liver lobule, narrowed and unrecognizable hepatic cords. By contrast, rats intervened with metformin and OBC showed regular liver cells and improved liver lobules and cords. Compared with rats interfered with metformin, the level of AST in all OBC groups decreased, and ALT in the OBC-l group decreased.

### Effect of OBC on Kidney Function

**Figure 8:**
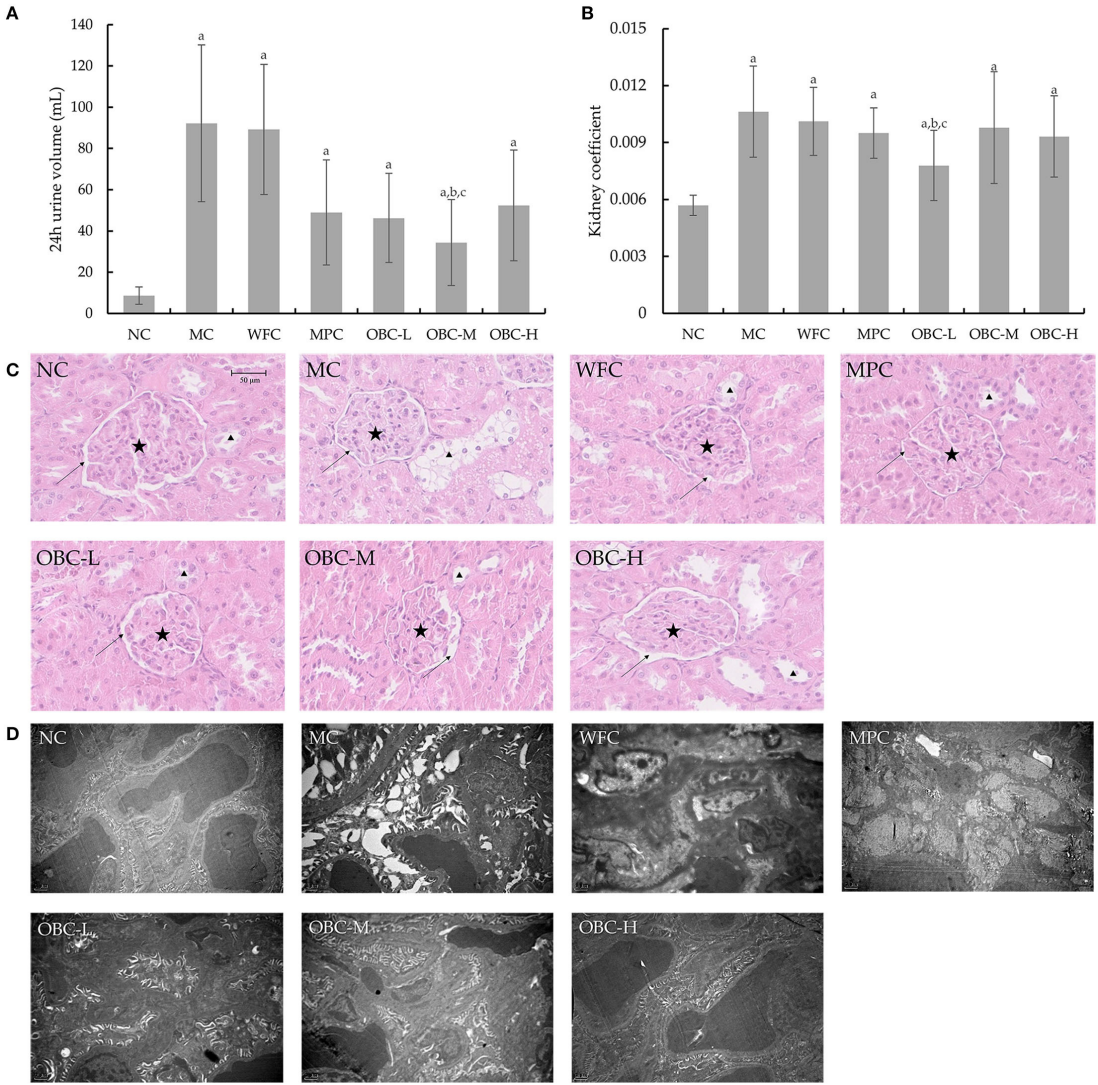
Effect of OBC on kidney function. **(A)** Six rats in each group were randomly selected to collect their 24 h urine at the end of the experiment. **(B)** The kidney coefficient is the ratio of kidney weight (g) to body weight (g). **(C)** HE staining was used to examining renal tissues, arrows indicate renal capsules, pentacles indicate glomeruli of kidneys and triangles indicate renal tubular epithelial cells. **(D)** Ultra microstructural changes of kidney in rats. NC (*n* = 10), MC (*n* = 9), WFC (*n* = 9), MPC (*n* = 8), OBC-L (*n* = 8), OBC-M (*n* = 10), OBC-H (*n* = 9), values are presented as mean ± SD. a *p* < 0.05 vs. NC rats, b *p* < 0.05 vs. MC rats, c *p* < 0.05 vs. WFC rats.

All diabetic rats showed a classic symptom of polyuria, as shown in Figure 8A. The 24-h urine volume of all diabetic rats was significantly higher than that of the rats in the NC group, and with the treatment of OBC, the 24-h urine volume of rats in the OBC-M group was markedly reduced, compared with rats in MC and WFC groups. Likewise, all diabetic rats showed an obviously increased kidney coefficient than normal rats, whereas rats in the OBC-L group showed a significantly decreased kidney coefficient compared with that in MC and WFC groups (Figure 8B).

As shown in Figure 8C, using HE staining, we found there were increases in glomerular volume and decreases of the renal capsule in renal tissues of MC, WFC, and MPC groups; in contrast, renal tissues in OBC groups were normal. Meanwhile, swelled renal tubular epithelial cells had been observed in MC and WFC groups, whereas less pathological changes showed in MPC and OBC groups. As shown in Figure 8D, compared with normal rats in the NC group, diabetic rats in the MC group showed increased glomerular volume, thickened capillary basement membrane, and vacuolar degeneration of renal tubular epithelial cells, indicating that significant renal tissue damage occurred in diabetic rats. Similar pathological damages occurred in rats in the WFC group. After the intervention of OBC, the renal damage of rats in each OBC group was improved. The structure of the glomerular basement membrane of rats intervened with OBC was clear and complete, and the arrangement of vascular endothelial cells was regular without obvious abnormalities.

### Effect of OBC on Ileal Intestinal Barrier

**Figure 9:**
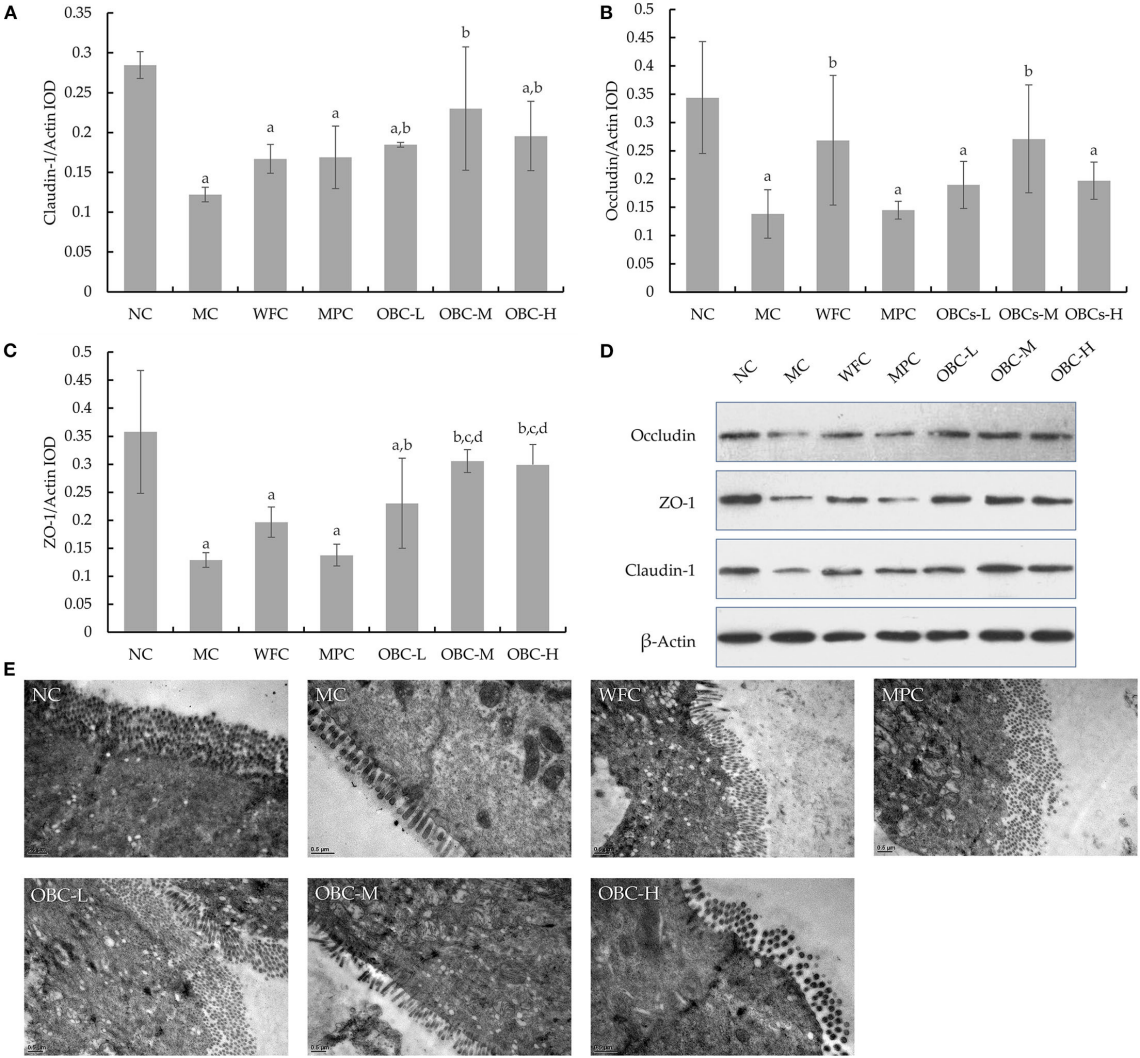
Ileal tight junction proteins expression in rats of each group. Statistical analysis for the expression of Claudin-1 **(A)**, ZO-1 **(B)**, and occludin **(C)**. Western blot images for the tight junction proteins expression **(D)**. Ultra-microstructural changes of ileal epithelium in rats **(E)**. The data were expressed as mean ± SD of each group. a *p* < 0.05 vs. NC rats, b *p* < 0.05 vs. MC rats, d *p* < 0.05 vs. MPC rats.

Compared with normal rats, the expression of Claudin-1, ZO-1, and Occludin was weakened significantly in diabetic rats in the MC group (Figure 9). All the rats that intervened with OBC showed enhanced expression of Claudin-1 and ZO-1, with the expression of Occludin enhanced in the OBC-M group. Besides, the expression of ZO-1 in the OBC-M and OBC-H groups was higher than that in the WFC group. Rats in the OBC-M and OBC-H groups showed higher ZO-1 expression than that of the MPC group. The morphology and ultrastructure of ileal villi in the NC group were normal, and no gap widening was observed. However, the ileal villi of diabetic rats were swollen, and the cell-tight junction structure was disordered. The ileal epithelial cell morphology and cell tight junction devices of rats treated with WFC and OBC were improved.

### Effect of OBC on Gut Microbiota

#### Operational Taxonomic Units

According to the sequence similarity (>97%), high-quality sequences were classified into multiple operational taxonomic units (OTUs) using QIIME to facilitate analysis. The number of OTUs detected in the cecal stool samples of rats in the seven test groups (NC, MC, WFC, MPC, OBC-L, OBC-M, OBC-H) were 507, 542, 562, 505, 516, 524, 522, respectively, whereas the number of unique OTUs was 6, 4, 12, 1, 2, 6, 6, respectively. There were 331 OTUs shared by seven groups.

#### Alpha Diversity Analysis

The observed species can reflect the actual number of OTUs observed. Chao1 is an estimator of phylotype richness, and the Simpson index of diversity reflects both the richness and community evenness. In this study, there was no significant difference found in the observed species (*p* = 0.26) and Chao1 index (*p* = 0.49), but there was a significant difference in the Simpson index (*p* = 0.02). MC group had higher evenness indexes [0.97 (0.98, 0.96)] than the NC group [0.96 (0.99, 0.93)], whereas each intervention group had a lower evenness index than MC group, WFC: 0.95 (0.97, 0.92), MPC: 0.91 (0.98, 0.84), OBC-L: 0.95 (0.98, 0.91), OBC-M: 0.93 (1.02, 0.85), OBC-H: 0.91 (0.99, 0.82). Indicators of alpha diversity were reported as the median ± IQR.

#### Beta Diversity Analysis

**Figure 10:**
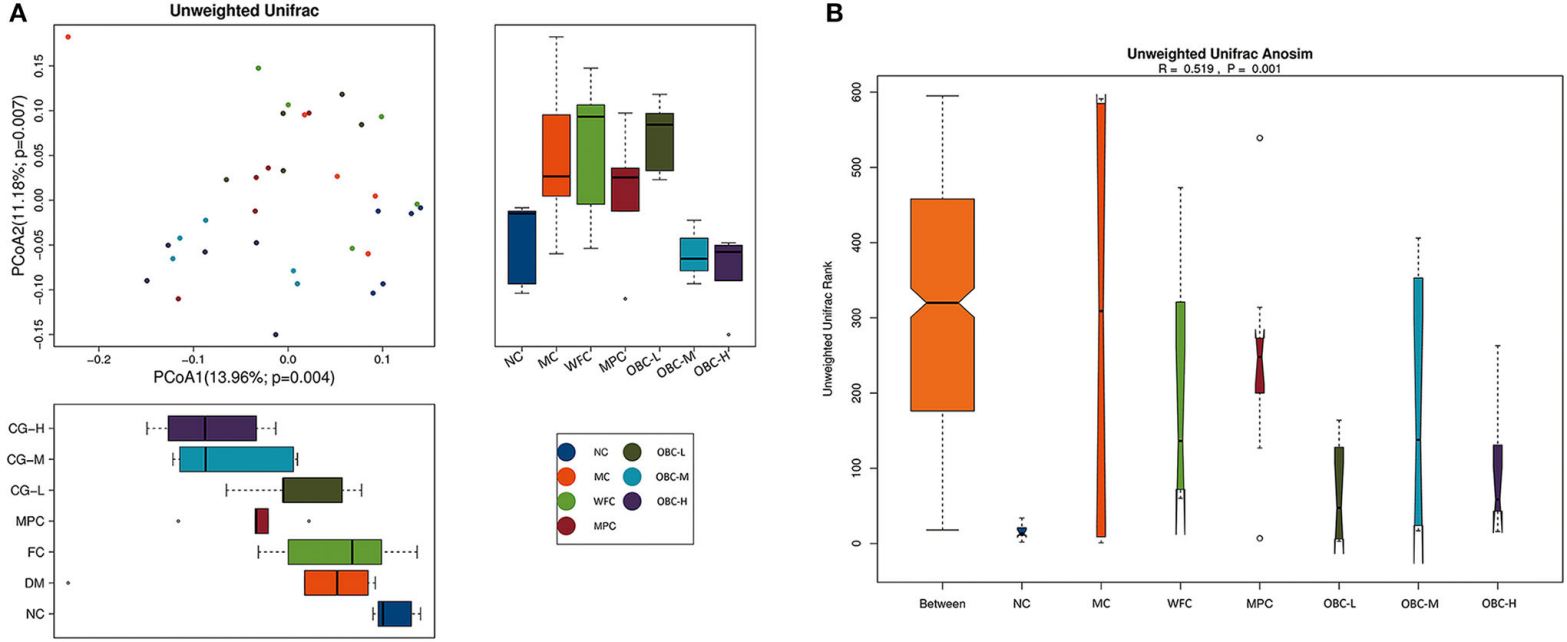
Effects on beta diversity. **(A)** PCoA of rats in each group. **(B)** Unifrac Anosim of rats in each group. R-value range (−1, 1).

Beta diversity analysis represents the extent of similarity between different microbial communities. Two principal components were extracted by PCoA. Figure 10 showed a clear separation between the fecal samples from the MC group and NC group, whereas OBC-M and OBC-H groups were close with the NC group. Percentage values at the axes indicated the contribution of the principal components to the explanation of total variance in the dataset. The figure showed that the percentages of variation explained by PC1 and PC2 were 13.96 and 11.18%, respectively.

In addition, ANOSIM demonstrated the differences in the gut microbiota among these groups. The intergroup differences among these groups were greater than the intragroup differences (*R* = 0.519, *p* = 0.001).

#### Classification Abundance Analysis

**Figure 11:**
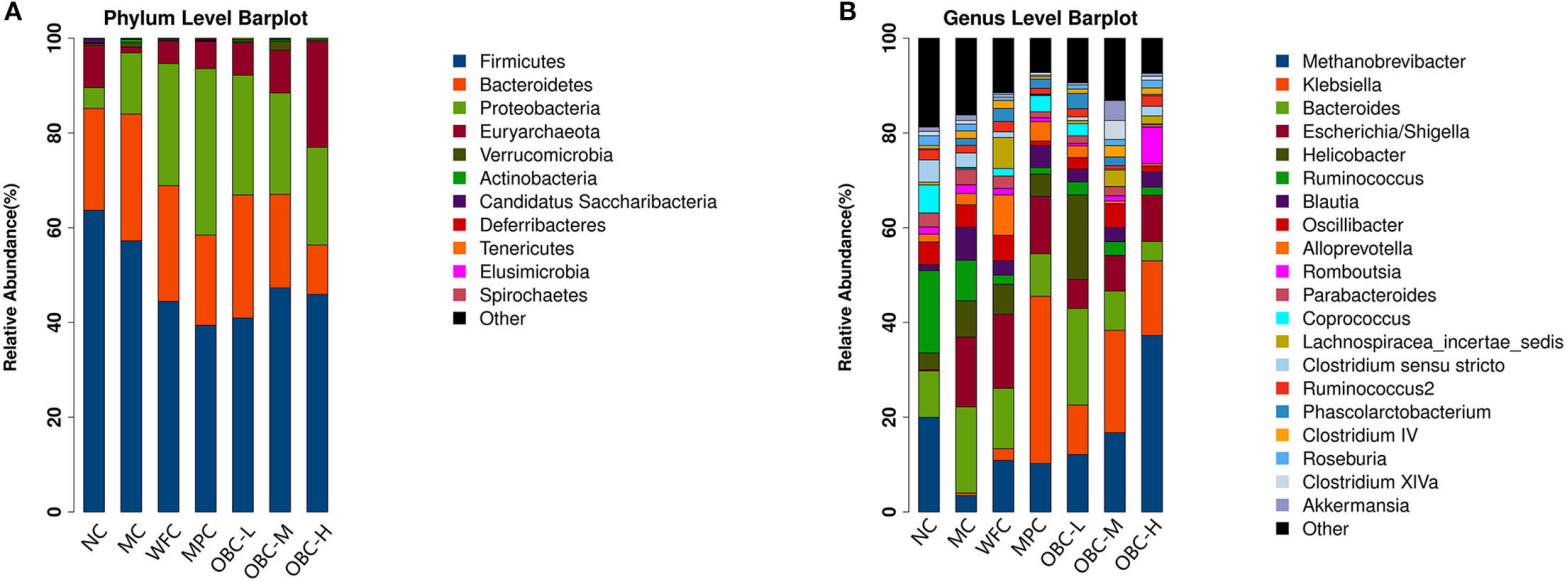
Microbial distributions at the phylum level **(A)** and the genus level **(B)** in the fecal samples from the seven groups. Each bar represents the microbiota composition of one group.

Figure 11 showed microbial distributions at the phylum and genus level in the fecal samples from the seven groups. Firmicutes and Bacteroidetes accounted for the largest proportion at the phylum level. At the genus level, Methanobrevibacter was the most proportional.

#### Axonomic Composition

To explore the specific bacterial taxa associated with OBC treatment, a LEfSe comparison of the gut microbiota among these seven groups was performed. The greatest difference in taxa from phylum to genus level was identified *via* the LDA score. Compared with normal rats, the Firmicutes phyla of diabetic rats is significantly reduced and the Actinobacteria phyla increased.

There were four significantly different phyla, with enrichment of Firmicutes in NC group, Actinobacteria in MC group, Proteobacteria in MPC group, and Euryarchaeota in OBC-H group.

Bacterial genus including Clostridium sensu stricto, Sutterella, Saccharibacteria genera incertae sedis, Clostridium XlVb, and Paraprevotella were found enriched in the NC group, Facklamia, Prevotella, Rhodococcus, Terrisporobacter, and Subdivision 5 genera incertae sedis were found enriched in the MC group, and Escherichia_Shigella, Oscillibacter, Flavonifractor, and Weissella were found enriched in the WFC group, Klebsiella was found enriched in MPC group. Helicobacter, Phascolarctobacterium, and Parasutterella were significantly intensified in the OBC-L group, Clostridium IV and Brevundimonas were significantly intensified in the OBC-M group and Methanobrevibacter, Methanosphaera, Enterococcus, Turicibacter, Eubacterium, and Anaerotruncus significantly intensified in the OBC-H group.

## Discussion

In this work, we demonstrated that as a staple food substitute, OBC has health-promoting effects on diabetes. To clarify these effects, we followed the changes in general state, blood glucose, and glucose tolerance of diabetic rats during the intervention of OBC and detected certain blood biomarkers. Diabetic rats intervened by OBC showed improved states, including increased body weight, decreased water take, and decreased urine volume. With decreased FBG, OGTT-AUC level as well as serum insulin level and HOMA-IR, rats in OBC groups showed improved glycometabolism and insulin resistance. Meliorated liver and kidney functions and antioxidant levels also showed that OBC can improve antioxidant capacity and reduce organ damage caused by diabetes. What is more, by promoting the expression of tight junction proteins in the intestine, OBC can improve the intestinal barrier. In the meantime, the consumption of OBC can increase the abundance of intestinal microbes and improve gut microbiota disturbance.

Diabetes has several common symptoms including polydipsia, polyuria, polyphagia, hyperglycemia, fatigue, and weight loss (14). STZ can cause pancreatic islet β-cell destruction, and is widely used experimentally to produce a diabetic rat model (10, 15, 16). In this work, after the injection of STZ at the end of the 6th week, while the FBG of the normal group rats remained in a normal range (5.45 ± 0.70 mmol/L), rats in each group injected by STZ showed significantly increased FBG, which ranged from 13.07 ± 4.92 mmol/L to 18.94 ± 3.71 mmol/L, indicating that the diabetic rat model was successfully established, which is consistent with previous studies on diabetic animal models (10). Besides, different from the continuous increase in body weight of the normal group, the weight of diabetic rats no longer gained or tend to decrease. The water consumption of diabetic rats continued to increase, as a symptom of polydipsia. Diabetic rats failed to keep a clean haircoat and showed a sign of fatigue. Elevated OGTT-AUC reflects the decrease of glucose tolerance in diabetic rats, the increase of HOMA-IR shows the formation of insulin resistance, with the raised FBG and insulin level, disorder of glucose metabolism in diabetic rats revealed. As shown in Table 3, the increased OGTT-AUC level of rats in the OBC-L group suggests that the intervention of OBC may be conducive to improve glucose tolerance in diabetic rats, which is in accordance with previous reports (10, 17). Relevantly, as shown in Figure 4, the consumption of OBC also led to an improvement in insulin metabolism and insulin resistance in diabetic rats, which in turn caused an improvement in glucose metabolism. Hyperglycemia-induced glycated molecules lead to increased oxidative stress in diabetic rats, which further resulted in the aggravation of multiple diabetic complications, including renal tubular injury (18, 19). In our study, diabetic rats intervened with OBC showed significantly reduced 24-h urine volume and kidney coefficient with ameliorated renal tissues and ultramicrostructure, whereas the serum SOD level markedly increased, indicating that OBC could reduce kidney injuries by enhancing antioxidant capacity. Our data also suggested that OBC may improve glucose metabolism by reducing oxidative stress. In previous studies, few of them had focused on the effect of interventions to reduce urine volume in diabetic rats, and our study showed that OBC could successfully reduce the urine volume of diabetic rats in 24 h, while reducing damages to the kidney caused by diabetes. Along with the result that rats in OBC groups showed less withered haircoat and less fatigue, these results showed an improvement in the quality of life of diabetic rats treated with OBC.

Several studies (8, 20–24) in relevant fields had demonstrated that oat oligopeptides, oat β-glucan, and buckwheat iminosugar d-fagomine could exert a hypoglycemic effect. Likewise, we tested the FBG of rats in each group weekly and did the oral glucose tolerance test biweekly, and the results confirmed that FBG and OGTT-AUC in OBC groups were lower than diabetic model rats and rats treated with wheat flour. Unlike our work, a previous experiment (10) showed that oat oligopeptides failed to cause changes in insulin in diabetic rats, and could improve insulin resistance and SOD level only if the oat oligopeptides were given in a high dose (2.00 g/kg body weight), though it may reduce the glucose level in diabetic rats. What's more, by contrast, in our work, after the kidney coefficient of diabetic rats increased, the intervention of OBC could reduce it. However, unlike the previous experiment, our work intervenes diabetic rats with the compound of two whole grains instead of a single ingredient of oat. Other functional ingredients of oat and buckwheat may be involved, considering the high level of rutin, quercetin, proteins, and minerals in buckwheat (20) and the high content of β-glucose and dietary fiber in oat, many of which have been proved to improve diabetes. In addition, our intervention of OBC had been implemented since the beginning of the experiment rather than after the injection of STZ. These differences in the intervention may account for the different results. However, while indicators of glucose metabolism such as FBG, OGTT-AUC and insulin, and proinflammatory cytokines, such as IL-6, in diabetic rats intervened with different doses of OBC were improved compared with rats in the MC group, suggesting that the intervention of OBC may correlate with the improvement of glucose metabolism and inflammatory reaction in diabetes, the phenotypes of each intervention group are not consistent throughout the work, and not all differences between the intervention groups and the MC group are statistically significant (*p* < 0.05). Thus, there was no clear dose-response relationship between OBC intervention and improvement of diabetes. This may be due to the complexity of ingredients in OBC and intervention pathways for diabetes. In addition, the limited sample size and grouping dose level in this work may also preclude the discovery of the dose-response relationship. Follow-up studies can explore a clearer dose-effect relationship by increasing the sample size and reducing the dosage gradient.

There is growing evidence that gut microbiota is closely associated with metabolic diseases including type 2 diabetes (25–28). Specific changes in the diversity of gut microbiota are one of the characteristics of diabetic patients and animals, and diet is one of the most influential factors that impacts the dynamic microbial community ecosystem (29). In the current work, the results from the 16s rDNA assay showed that OBC had a significant effect on gut microbiota in diabetic rats. While the Simpson index was increased in the MC group, diabetic rats treated with OBC showed a lower Simpson index, indicating that OBC treatment could make gut microbiota show a lower evenness. What's more, Beta diversity analysis showed that the gut microbial community of MC rats was significantly different from that of normal rats, whereas the gut microbial community of OBC-M and OBC-H rats was more similar to that of normal rats, indicating that OBC may exert to alter microbiota composition to make it more resembling of microbiotas of healthy rats.

Reasons for OBC impacting gut microbiota may be related to its components. Higher Parasutterella abundance was observed in the OBC-L group. Though there is insufficient knowledge on the relationship between specific strains and dietary components, evidence has shown that complex carbohydrates may be involved. Yuan et al. (30) found that polysaccharides could increase the abundance of Parasutterella. Fermented by gut bacteria, various complex carbohydrates could be the major fuel source of the microbiota (31, 32) to exert a major impact to promote the growth of the probiotic and positively influence probiotic-enterocyte interaction (33). Besides, unique phytochemicals contained in OBC, such as buckwheat phenolic acids, ferulic acid, and oat avenanthramides, have been shown to favor specific bacterial species (32–34).

The underlying mechanisms of gut microbiota impacted by OBC regulating host metabolism may include favorable changes in bacterial composition and/or activity, improvement of intestinal barrier function (35), productions of short-chain fatty acids (SCFAs), and inhibition of inflammation (36, 37). Certain species of probiotics like lactobacilli possess potent alpha-glucosidase inhibitory activity that prevents the breakdown of complex carbohydrates and reduces postprandial hyperglycemia (38), while other bacteria like Firmicutes are usually involved in the transport of nutrients and facilitates the absorption and fermentation of SCFA, which are essential for maintaining insulin sensitivity (39) and reducing local inflammation by regulating the expression of proinflammatory cytokines as TNF-α and IL-6 through the activation of macrophages and dendritic cells (40). TNF-α and IL-6 are important inflammatory factors involved in inflammatory response, often used as diagnostic indicators of diabetes and related complications such as diabetic nephropathy. On the contrary, potentially pathogenic bacteria as Rhodococcus could inhibit the expression of tight junction proteins like occludin, claudin-1, and ZO-1, while it produces metabolites that can destroy the epithelial barrier and increase gut permeability (38, 41). Thus, microbiota products such as lipopolysaccharide can drive low-grade inflammation, which had long been recognized as a potential cause of insulin resistance (36, 42). However, unlike the commonly reported findings (32, 43), no intergroup difference was found in bifidobacteria and lactobacilli in this study. This may be due to the strain of experimental animals, feeding diets, and geographical location (44).

In addition, this study shows that OBC can reduce inflammation, moderate liver damage, and improve the intestinal barrier, whereas metformin, which is a commonly used drug for diabetes treatment, cannot. It is noteworthy that there was no difference in food taken, this suggesting the palatability of OBC. Besides, compared with medication, people are also more receptive to OBC, common foods in daily life, and thus OBC is a more suitable potential food for diabetes prevention and long-term intervention.

Although this work could provide new insights to explore the diet intervention on diabetes, it has several limitations. First, in our work there was only one compounding ratio of buckwheat and oat, and more compounding ratios may enhance the experimental evidence. Second, although this work shows that the consumption of OBC is closely related to the improvement of diabetes and gut microbiota ameliorating, further research, such as fecal bacteria transplantation, is needed to decipher the causal relationship involving bacterial disequilibrium and diabetes and to explore key bacteria which may play an important role in the intervention. Third, follow-up studies are needed to support the improvement of OBC on glucose tolerance in diabetes, and to explore the recommended dose of OBC for the specific dietary pattern.

## Conclusion

The results revealed that as a staple food substitute, OBC could exert promoting effects on diabetic rats. By improving antioxidant capacity, reducing inflammation, and improving the imbalance of intestinal microbiota, the glucose and lipid metabolism and insulin resistance of diabetic rats have been improved. Meanwhile, by improving a variety of tissues and organs in the diabetic state, OBC may have the potential to prevent diabetic complications. OBC can be used as a potential staple food substitute in dietary patterns that are suitable for the Chinese.

## Data Availability Statement

The original contributions presented in the study are included in the article/Supplementary Material, further inquiries can be directed to the corresponding author.

## Ethics Statement

The animal study was reviewed and approved by Ethics Committee of Peking University.

## Author Contributions

SL and JW: conceptualization and methodology. SL: formal analysis and writing original draft preparation. SL, CH, XL, XY, HM, and XZ: investigation. YX: resources. SL and XY: data curation. MX and JW: writing review and editing. YL and JW: supervision. JW: project administration and funding acquisition. All authors contributed to the article and approved the submitted version.

## Funding

This research was funded by the Ministry of Science and Technology of the People's Republic of China, Grant Number 2017YFD0401202.

## Conflict of Interest

The authors declare that the research was conducted in the absence of any commercial or financial relationships that could be construed as a potential conflict of interest.

## Publisher's Note

All claims expressed in this article are solely those of the authors and do not necessarily represent those of their affiliated organizations, or those of the publisher, the editors and the reviewers. Any product that may be evaluated in this article, or claim that may be made by its manufacturer, is not guaranteed or endorsed by the publisher.
