# Supplementary material for: As a Staple Food Substitute, Oat and Buckwheat Compound Has Health-Promoting Effects for Diabetic Rats
Source: Front Nutr. 2021 Dec 24;8:762277. doi: 10.3389/fnut.2021.762277 (PMC8740054; doi:10.3389/fnut.2021.762277)
Supplement: Supplementary file 1 [file Data_Sheet_1.docx]

Supplementary Material

# Supplementary Figures


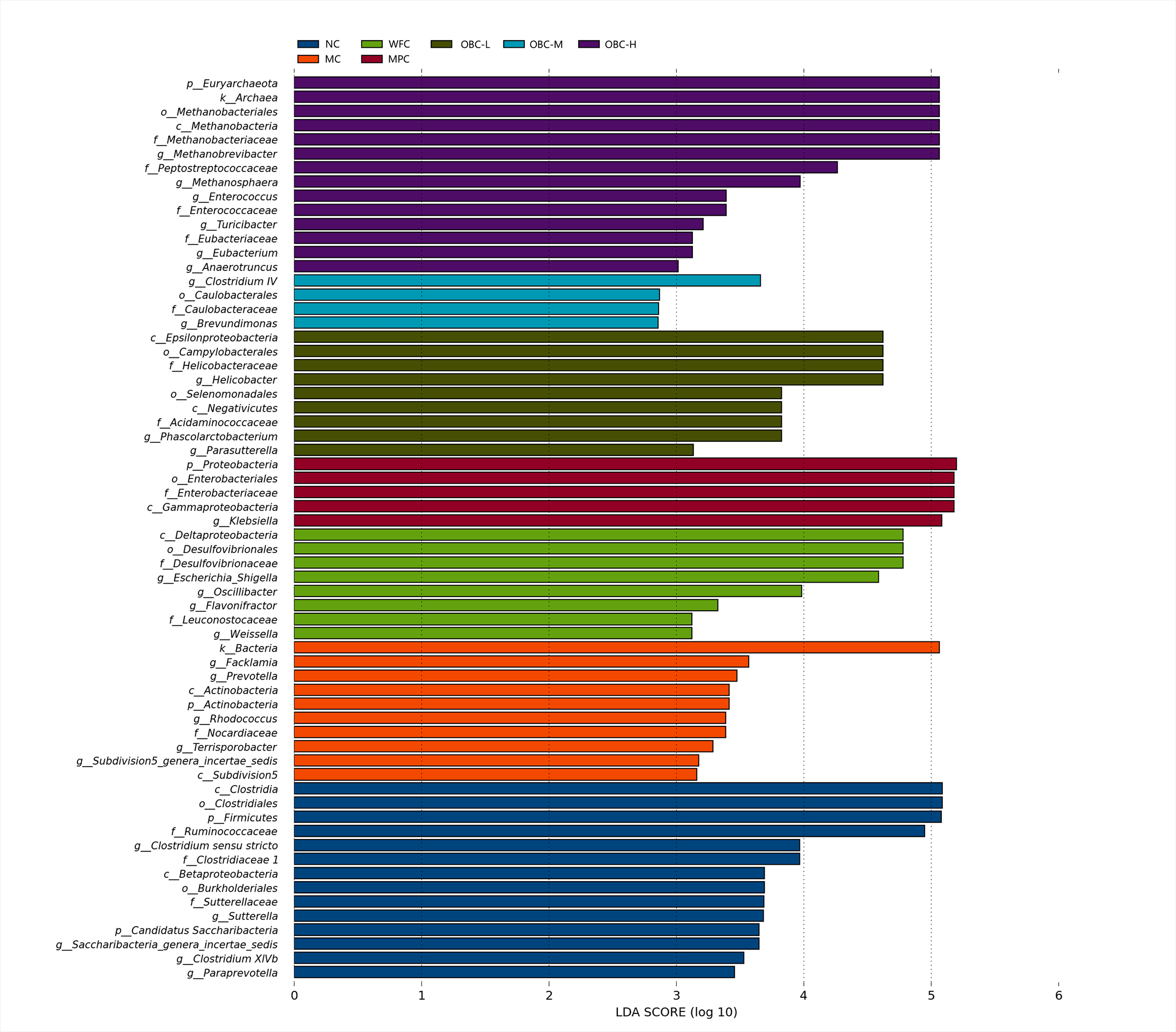


**Supplementary Figure 1.** The most differentially abundant taxa among the 7 groups were identified through the LDA score which was generated from LEfSe analysis (phylum to genus: p, phylum; c, class; o, order; f, family; g, genus). (b) The enriched taxa in each group were represented in Cladogram. The central point represents the root of the tree (Bacteria), and each ring represents the next lower taxonomic level (phylum to genus: p, phylum; c, class; o, order; f, family; g, genus). The diameter of each circle represents the relative abundance of the taxon.


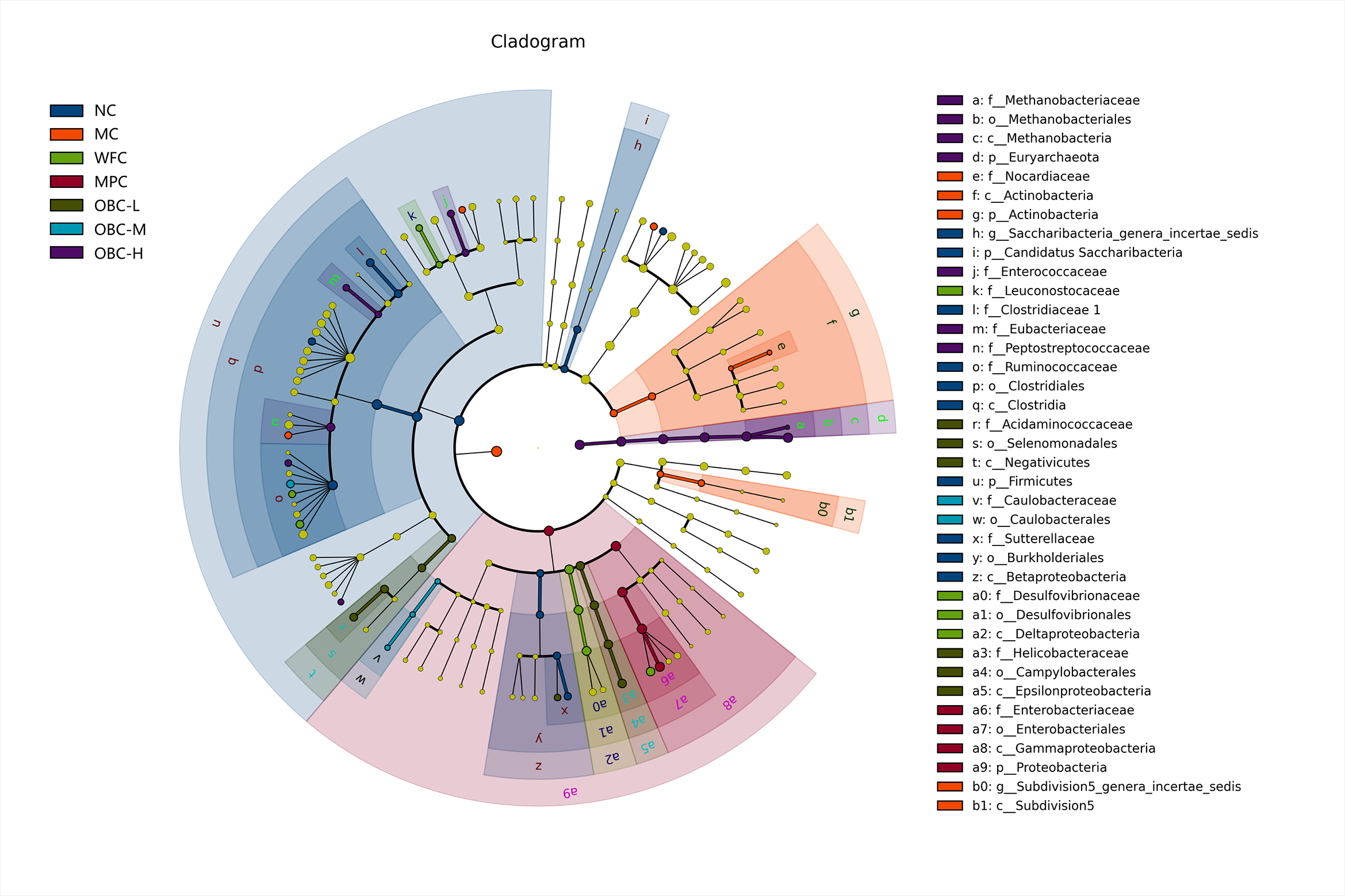


**Supplementary Figure 2.** The enriched taxa in each group were represented in Cladogram. The central point represents the root of the tree (Bacteria), and each ring represents the next lower taxonomic level (phylum to genus: p, phylum; c, class; o, order; f, family; g, genus). The diameter of each circle represents the relative abundance of the taxon.

# Supplementary Table

**Supplementary Table 1.** Feed ingredients of each group (g/kg)

| Group | Casein, 80 Mesh | L-Cystine | Corn Starch | wheat flour | OBC | Maltodextrin 10 | Sucrose | Cellulose, BW200 | Soybean Oil | Lard | Mineral Mix S10026 | DiCalcium Phosphate | Calcium Carbonate | Potassium Citrate, 1 H2O | Vitamin Mix V10001 | Choline Bitartrate | FD&C Yellow Dye #5 | FD&C Red Dye #40 | FD&C Blue Dye #1 |
| --- | --- | --- | --- | --- | --- | --- | --- | --- | --- | --- | --- | --- | --- | --- | --- | --- | --- | --- | --- |
| NC | 189.6 | 2.8 | 298.6 | 0.0 | 0.0 | 33.2 | 331.7 | 47.4 | 23.7 | 19.0 | 9.5 | 12.3 | 5.2 | 15.6 | 9.5 | 1.9 | 0.0 | 0.0 | 0.0 |
| MC | 233.1 | 3.5 | 84.8 | 0.0 | 0.0 | 116.5 | 201.4 | 58.3 | 29.1 | 206.8 | 11.7 | 15.1 | 6.4 | 19.2 | 11.7 | 2.3 | 0.0 | 0.1 | 0.0 |
| WFC | 233.1 | 3.5 | 0.0 | 159.3 | 0.0 | 42.1 | 201.4 | 58.3 | 29.1 | 206.8 | 11.7 | 15.1 | 6.4 | 19.2 | 11.7 | 2.3 | 0.0 | 0.1 | 0.0 |
| MPC | 233.1 | 3.5 | 84.8 | 0.0 | 0.0 | 116.5 | 201.4 | 58.3 | 29.1 | 206.8 | 11.7 | 15.1 | 6.4 | 19.2 | 11.7 | 2.3 | 0.0 | 0.1 | 0.0 |
| OBC-L | 233.1 | 3.5 | 45.0 | 0.0 | 39.9 | 116.5 | 201.4 | 58.3 | 29.1 | 206.8 | 11.7 | 15.1 | 6.4 | 19.2 | 11.7 | 2.3 | 0.0 | 0.1 | 0.0 |
| OBC-M | 233.1 | 3.5 | 5.1 | 0.0 | 79.7 | 116.5 | 201.4 | 58.3 | 29.1 | 206.8 | 11.7 | 15.1 | 6.4 | 19.2 | 11.7 | 2.3 | 0.0 | 0.1 | 0.0 |
| OBC-H | 233.1 | 3.5 | 0.0 | 0.0 | 159.3 | 42.1 | 201.4 | 58.3 | 29.1 | 206.8 | 11.7 | 15.1 | 6.4 | 19.2 | 11.7 | 2.3 | 0.0 | 0.1 | 0.0 |

NC: normal control group, MC: model control group, WFC: wheat flour control group, MPC: metformin positive control group, OBC-L: low-dose group, OBC-M: medium-dose group, OBC-H: high-dose group.
